# Biomarkers for parkinsonian disorders in CNS-originating EVs: promise and challenges

**DOI:** 10.1007/s00401-023-02557-1

**Published:** 2023-04-04

**Authors:** Suman Dutta, Simon Hornung, Hash Brown Taha, Gal Bitan

**Affiliations:** 1 International Institute of Innovation and Technology, New Town, Kolkata, India; 2 Division of Peptide Biochemistry, TUM School of Life Sciences, Technical University of Munich, Freising, Germany; 3 Department of Integrative Biology and Physiology, University of California Los Angeles, Los Angeles, CA USA; 4 Department of Neurology, David Geffen School of Medicine at UCLA, University of California Los Angeles, 635 Charles E. Young Drive South/Gordon 451, Los Angeles, CA 90095 USA; 5 Brain Research Institute, University of California Los Angeles, Los Angeles, CA USA; 6 Molecular Biology Institute, University of California Los Angeles, Los Angeles, CA USA

**Keywords:** Parkinson’s disease, Multiple system atrophy, Lewy bodies, L1CAM, Neurodegenerative diseases, Neurons, Astrocytes, Microglia, Oligodendrocytes, Extracellular vesicles, Exosomes

## Abstract

Extracellular vesicles (EVs), including exosomes, microvesicles, and oncosomes, are nano-sized particles enclosed by a lipid bilayer. EVs are released by virtually all eukaryotic cells and have been shown to contribute to intercellular communication by transporting proteins, lipids, and nucleic acids. In the context of neurodegenerative diseases, EVs may carry toxic, misfolded forms of amyloidogenic proteins and facilitate their spread to recipient cells in the central nervous system (CNS). CNS-originating EVs can cross the blood–brain barrier into the bloodstream and may be found in other body fluids, including saliva, tears, and urine. EVs originating in the CNS represent an attractive source of biomarkers for neurodegenerative diseases, because they contain cell- and cell state-specific biological materials. In recent years, multiple papers have reported the use of this strategy for identification and quantitation of biomarkers for neurodegenerative diseases, including Parkinson’s disease and atypical parkinsonian disorders. However, certain technical issues have yet to be standardized, such as the best surface markers for isolation of cell type-specific EVs and validating the cellular origin of the EVs. Here, we review recent research using CNS-originating EVs for biomarker studies, primarily in parkinsonian disorders, highlight technical challenges, and propose strategies for overcoming them.

## Introduction

Neurodegenerative diseases affect one in six people and unlike other major deadly diseases, such as many types of cancer, COVID-19, or AIDS, for which multiple treatment options exist, there are almost no disease-modifying therapies for neurodegenerative diseases [22]. Neurodegenerative diseases also suffer from high rates of misdiagnosis [17, 70, 105, 154, 158]. Parkinson’s disease (PD) and atypical parkinsonian syndromes, including the synucleinopathies dementia with Lewy bodies (DLB) and multiple system atrophy (MSA), and the tauopathies progressive supranuclear palsy (PSP) and corticobasal syndrome (CBS), are neurodegenerative diseases characterized by a movement disorder, often autonomic dysfunction, and in some cases, dementia. These diseases differ pathologically, yet due to symptom overlap, they often are misdiagnosed, particularly in the early stages when patients are likely to consult general clinicians or neurologists rather than movement disorder specialists [2, 17]. Misdiagnosis causes high levels of anxiety to patients, families, and caregivers and is a major impediment to conducting successful clinical trials.

Several conditions, such as isolated REM-sleep behavior disorder (iRBD) and pure autonomic failure (PAF) are known to be strong risk factors for the development of PD, DLB, or MSA and could be utilized for stratifying patients into clinical trials, yet it is difficult to predict based on clinical measures whether a particular patient will phenoconvert into one of these central nervous system (CNS) synucleinopathies and into which specific disease, hindering the stratification efforts. Objective biomarkers could alleviate these issues, allow inclusion of prodromal or early-stage patients who are most likely to benefit from the therapy, and increase the likelihood of trial success. Thus, there is an urgent need to discover, develop, and validate sensitive and specific biomarkers for parkinsonian disorders. Reliable biomarkers not only will allow making an accurate diagnosis at an early stage, but also are crucial for monitoring treatment outcomes. However, due to the inaccessibility of the CNS, discovery and measurement of such biomarkers is challenging.

Common approaches to CNS disease biomarkers include various modalities of brain imaging [26, 34, 71, 117, 180] and analysis of biomarkers in the cerebrospinal fluid (CSF) [49, 53, 118, 196]. Both approaches are useful, yet suffer from important shortcomings. CNS imaging often does not have the required accuracy and sensitivity and tends to be expensive. Some of the imaging techniques considered most useful, such as positron emission tomography (PET) or single photon emission computed tomography (SPECT), are not available outside of major hospitals. GE Healthcare’s DaTscan, which measures the degeneration of striatal dopaminergic neurons, has been the biomarker of choice for parkinsonian syndromes, including in recent clinical trials. However, DaTscan has a limited ability to distinguish among parkinsonian syndromes, in current clinical settings it is used as a qualitative but not a quantitative biomarker and, importantly, the results are affected by medications used for PD treatment, complicating data interpretation.

CSF analysis measures CNS analytes with high accuracy, yet the necessary invasive lumbar puncture is refused by many patients. It is particularly challenging to use CSF biomarkers in clinical trials that require monitoring the outcome at multiple time points. An important development for the diagnosis of parkinsonian disorders is the ability to perform seed-amplification reactions using techniques called protein misfolding cyclic amplification (PMCA) [163] or real-time quaking-induced conversion assay (RT-QuIC) [188]. These assays were developed first in the prion field and later expanded to other neurodegenerative proteinopathies, including synucleinopathies [75]. Using seed amplification, Shahnawaz et al. demonstrated that CSF samples from patients with PD could be distinguished from those of patients with MSA with high accuracy [151], offering hope that the same technique could be used in the future in samples obtained using less invasive means, such as serum or plasma.

Recently, ultrasensitive techniques, such as electrochemiluminescence ELISA (ECLIA) [165, 195] and single-molecule array (Simoa) [161], have allowed analysis of promising biomarkers directly in plasma or serum, e.g., neurofilament light chain (NfL), a highly useful biomarker of neurodegeneration, or the protein tau phosphorylated at Thr 217 (pT217-tau) as a specific marker for Alzheimer’s disease (AD) [16, 135, 141]. Although serum/plasma NfL is a useful biomarker also for parkinsonian disorders, disease-specific, blood-based biomarkers for these diseases have yet to be identified. The very low blood concentration of molecules originating in the CNS and the vulnerability of most such potential biomarkers to degradation in the blood make discovering and developing blood-based biomarkers for parkinsonian disorders challenging.

An alternative approach is the analysis of biomarkers in extracellular vesicles (EVs) originating in the CNS and isolated from the blood [78, 127]. One way the CNS communicates with the rest of the body is by ferrying EVs through the blood–brain barrier (BBB) to distant cells [66]. The capture of these EVs from a blood sample and analysis of their content provide a window into biochemical changes in the brain. The advantages of this approach compared to direct measurement of biomarkers in plasma or serum include facilitated transfer of the target biomarkers inside EVs through the BBB and their protection from enzymatic degradation, which translates into a larger variety of biomarkers in the EVs compared to those measured directly in blood. In addition, this route increases the overall sensitivity of the downstream assay, because other blood contaminants present in large quantities do not mask the minute, yet crucial signals coming from the CNS. Importantly, the biomarkers of interest can be measured separately in EVs originating in different CNS cell types. We have shown recently that this can be crucial for obtaining high sensitivity and specificity of candidate diagnostic biomarkers, where the biomarker levels in each cell type alone did not provide sufficient diagnostic power [47, 169].

**Fig. 1:**
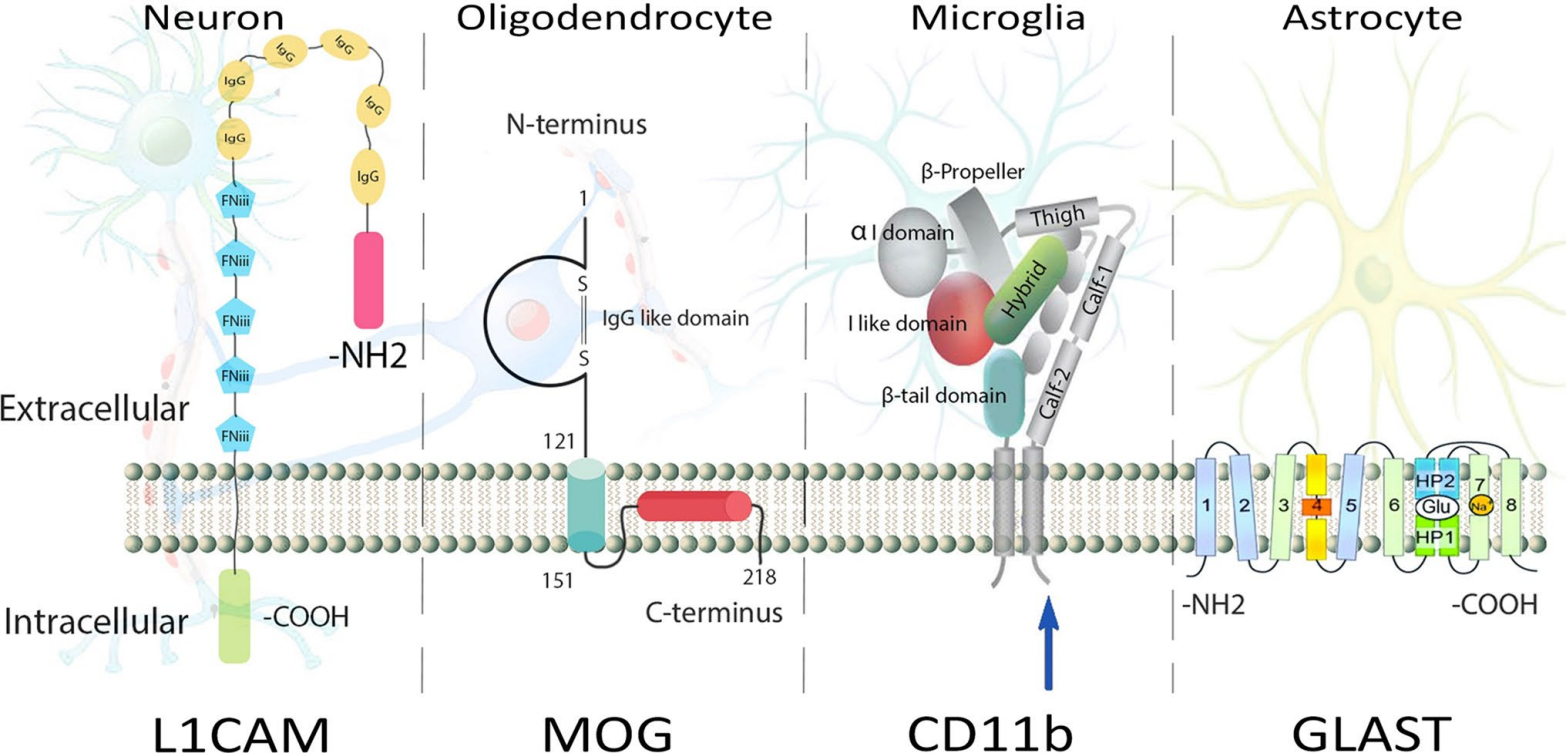
Schematic structures of commonly used marker proteins for IP of CNS-originating EVs. L1CAM contains six IgG-like domains and five fibronectin type III repeats, followed by a transmembrane part and a conserved cytoplasmic tail [207]. The model of MOG membrane topology is based on the one described by Kroepfl et al. [100]. The structure of CD11b is shown in the context of a heterodimeric integrin. The other subunit is CD18 [124]. The depicted membrane topology of GLAST is a model consisting of eight transmembrane domains and two hairpin loops [203]

A crucial step in this methodology is the isolation and separation of the correct population of EVs—those originating in the CNS—from all other EVs in the blood, which represent virtually every cell in the body. This is done by immunoprecipitation (IP) of the EVs using selective markers of brain cells expected to be displayed on the EV surface, such as neuronal cell adhesion molecule (NCAM) or L1 cell adhesion molecule (L1CAM, CD171) for putative neuronal EVs (nEVs) [54, 153], glutamate-aspartate transporter (GLAST) for astroglial EVs (aEVs) [63], CD11b or transmembrane protein 119 (TMEM119) for microglial EVs (mEVs) [40, 101], and myelin oligodendrocyte glycoprotein (MOG) for oligodendroglial EVs (oEVs) [47] (Fig. 1). A current challenge in the field is validation that EVs immunoprecipitated using such markers indeed originated in these CNS cells.

The difficulty in validating the cellular origin of EVs has caused concerns regarding their actual specificity. Recently, doubts have been raised particularly regarding the use of L1CAM, a heterogeneous protein that exists in both soluble and membrane-bound forms, for the isolation of nEVs [132]. These concerns prompted the International Society for Extracellular Vesicles (ISEV) together with the Michael J. Fox Foundation for Parkinson’s Research to hold a joint session, entitled “The L1CAM Controversy: Pulling Down Consensus” in the spring of 2021, in which this and several other conceptual and methodological topics related to the measurement of biomarkers in CNS-originating EVs were discussed. These topics are the focus of our review in which we summarize the current state of the field and propose directions for reconciling differences and moving forward.

## Extracellular vesicles as a source of objective biological markers for CNS disorders

EVs are a heterogeneous population of biological vesicles enclosed by a lipid bilayer, including exosomes, microvesicles, oncosomes, and apoptotic bodies [42, 145]. A more recently discovered subpopulation of EVs termed 'exomeres' comprises non-membranous nanovesicles with a diameter ≤ 50 nm [8]. Virtually all eukaryotic cell types, including those of the CNS, produce and release EVs. Exosome biogenesis involves the invagination of the endosomal membrane to form multivesicular bodies (MVBs) containing intraluminal vesicles. The MVBs subsequently fuse with the cell membrane to release the intraluminal vesicles into the extracellular space, at which point these vesicles are termed exosomes [27]. In contrast, microvesicles, also called ectosomes, evaginate directly from the plasma membrane [27]. The diameter of exosomes ranges from 30 to 200 nm [199] whereas microvesicles are typically larger, ranging from 200 to 1000 nm in diameter. Both vesicle types coexist in bodily fluids and conditioned cell culture medium [121, 181]. The nomenclature of EVs has been a matter of debate as the number of publications in this field has increased exponentially in the last two decades [194]. According to ISEV guidelines [114, 172], “extracellular vesicle” is favored as a generic term for biological particles released from cells that are enclosed by a lipid bilayer and unable to replicate and therefore we use this term here.

EVs carry various cargoes including messenger RNA (mRNA), non-coding RNAs (ncRNAs), lipids, and proteins. Although they were originally believed to be a disposal mechanism of unwanted biological material [90, 136], it became evident later that they play major roles in intercellular communication and signaling pathways [27, 178, 200]. In the CNS, EVs contribute to the maintenance of myelination, trophic support of neurons, synaptic plasticity, and antigen presentation [98, 109, 173]. CNS-originating EVs have been isolated successfully from human plasma [54, 153], serum [47], and saliva [144] suggesting that they could serve as rich sources of biomarkers using minimally invasive means.

**Fig. 2:**
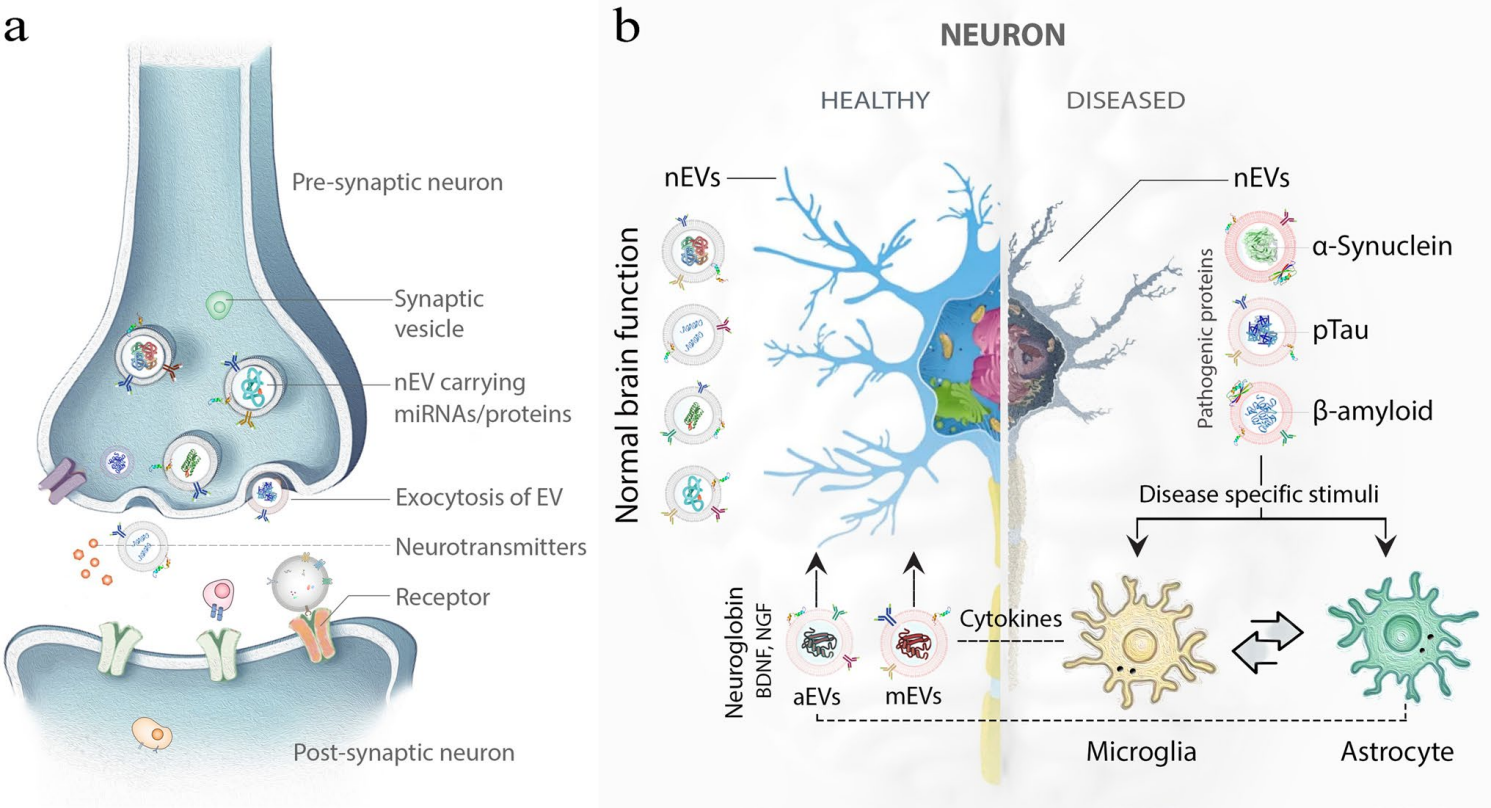
Involvement of EVs in the CNS microenvironment. **a** EVs originating in, and released by, neurons are taken up by neighboring cells, including other neurons, spreading information across the CNS and modulating synaptic activities both anterogradely and retrogradely. EVs also may act as novel neurotransmitters [69]. Under pathological conditions, EVs from stressed or damaged neurons may propagate disease pathology. **b** EVs contain cell- and cell state-specific cargo representing the parent cells’ biochemical environment. EVs produced by healthy neurons may contain subsets of biomolecules required for normal cellular function including mediators of regular intercellular communications. In contrast, EVs originating from ailing neurons of proteinopathy brains may harbor elevated concentration levels of pathogenic proteins, such as α-syn, p-tau, and/or Aβ, triggering inflammatory responses by microglia and astrocytes

In the context of parkinsonian disorders and other neurodegenerative proteinopathies, EVs may be a double-edged sword. They facilitate the expulsion of pathologic proteoforms when the cellular clearance mechanisms, including the ubiquitin–proteasome system (UPS) and autophagy–lysosomal pathway (ALP), become insufficient [6, 60, 83, 123]. Simultaneously, because the EVs may be taken up by recipient cells (Fig. 2a), they are also important mediators of the pathology spread, transporting key pathological protein oligomers and aggregates among CNS cells (Fig. 2b) [41, 57, 173]. Thus, the presence of various amyloidogenic proteins, such as amyloid β-protein (Aβ), α-synuclein (α-syn), tau, different phosphorylated forms of Tau (p-Tau), and TAR DNA-binding protein 43 kDa (TDP-43) has been demonstrated in EVs [52, 69, 107, 143]. Importantly, CNS-originating EVs cross the BBB and can be isolated from blood products [47, 54, 133, 153, 192], providing a “window” into biochemical changes in the CNS.

Biomarkers are objectively measured indicators of normal biological processes, pathogenic transformations of biological processes, and responses to intervention [21]. Biomarkers always should be qualified for a specific context in which they are used [125], such as: (1) predictive biomarkers for evaluating disease probability at pre-clinical phases; (2) diagnostic biomarkers for differential disease identification; (3) prognostic biomarkers to assess the probability of healing; (4) progression biomarkers for monitoring disease severity and progression over time; and (5) treatment-effect biomarkers for measuring the efficiency of therapeutic intervention [111, 119, 125]. Most studies using CNS-originating blood EVs have focused on the differential diagnosis of patient groups of one or more neurodegenerative disease(s) compared to healthy control subjects and each other [47, 54, 133, 153, 166, 212]. In some cases, changes in the biomarkers have been shown to correlate with disease progression cross-sectionally, or more rarely longitudinally. Due to the lack of FDA-approved disease-modifying therapy for most neurodegenerative diseases, treatment-effect biomarkers are still relatively rarely studied [12, 115]. In a recent study, Palma et al. investigated the safety and efficacy of sirolimus in patients with MSA and analyzed several biomarker modalities, including α-syn in CNS-originating EVs, in the trial participants [134].

## Isolation and analysis of CNS-originating cell type-specific EVs as a source of biomarkers for neurodegenerative disorders

It is not yet known if specific processes, e.g., glymphatic efflux, are involved in the transportation of EVs out of the brain, or whether different mechanisms exist for the excursion of CNS-originating EVs into biofluids, such as blood or urine. EVs can be isolated from bodily fluids using different methodologies, including ultrafiltration/ultracentrifugation, microfluidic arrays, size exclusion-based methods, immunoaffinity capture, or by using commercially available polymer-based EV precipitation kits [43, 45, 47, 199, 209]. When the goal is to study neurodegenerative diseases, ideally, the EVs studied would be those originating in the CNS. To achieve this goal, researchers have used two different strategies: capturing the CNS-originating EVs directly from the fluid by IP, or a two-step process in which first all the EVs are isolated from the biofluid, followed by enrichment of CNS-originating EVs by IP. In both strategies, the CNS-originating EVs are immunoprecipitated using bead-conjugated antibodies against cell-specific markers (Fig. 1). To improve the enrichment and reduce contamination by highly abundant blood-resident EVs, a negative selection step, for example using anti-CD45/CD61 antibody-coated beads, as shown by Ko et al. [94], could remove the majority of non-CNS-EVs before IP using CNS cell-specific markers.

### Neuron-originating EVs (nEVs)

nEVs have many roles in the CNS, including mediating neuron–glia communication, neuroprotection, neuroregeneration, synaptic plasticity, and under disease conditions, dissemination of pathological biomaterials [36, 81, 103, 113]. Because neurons are the cell type most affected in neurodegenerative proteinopathies, nEVs have been the focus of most biomarker studies using this strategy. Two research groups pioneered this field, the Zhang group at the University of Washington, Seattle, and the Goetzl group at the University of California, San Francisco. Both groups used anti-L1CAM and/or anti-NCAM antibodies to IP nEVs from blood samples [54, 153] (Table 1).

**Table 1:** Methods used for nEV enrichment from blood in selected recent studies

Group	Antibody	Support system	EV isolation method	References
Goetzl	Anti-NCAM (ERIC1); anti-L1CAM (clone 5G3)	Streptavidin-Plus UltraLink resin	EV precipitation using ExoQuick, then resuspension and incubation with the biotinylated antibody for 1 h at 4 °C. Capture of antibody–EV complexes by addition of streptavidin-agarose resin, centrifugation, and resuspension of the pellet	[54, 61–63, 127]
Pulliam	Anti-L1CAM (clone 5G3)	Streptavidin-Plus UltraLink resin	Plasma coagulation proteins were removed using thrombin, followed by centrifugation at 3,000×g for 20 min. EV precipitation using ExoQuick, resuspension, and incubation with the biotinylated antibody for 1 h at 4 °C, followed by the capture of labeled EVs with streptavidin-conjugated agarose beads. nEV–resin complexes were washed and nEVs were released from the beads using 50 mM glycine–HCl, pH 3	[142, 167]
Zhang	Anti-L1CAM (clone UJ127)	M-270 Epoxy Dynabeads^®^	Centrifugation of plasma samples at 2,000×*g* for 15 min, followed by 12,000×*g* for 30 min, and dilution 1:3 of the supernates with PBS, pH 7.4 EV capture by incubation of diluted plasma with antibody-coated Dynabeads^®^ for 24 h at 4 °C with gentle rotation. Washing of bead–EV complexes and elution using fixing buffer or lysis of captured EVs	[153, 174, 208]
Tofaris	Anti-L1CAM (clone UJ127)	Poly(carboxybetaine methacrylate) (pCBMA)-coated beads	Serum samples were cleared by multi-step sequential centrifugation and incubated overnight at 4 °C with pCBMA-coated beads pre-conjugated with anti-L1CAM antibody Magnetic separation and washing of bead–EV conjugates before lysis	[86, 87]
Rissman	Anti-L1CAM (clone 5G3)	Streptavidin-Plus UltraLink resin	EV precipitation using ExoQuick, then resuspension and incubation with the biotinylated antibody for 1 h at 20 °C. The capture of antibody–EV complexes by the addition of streptavidin-agarose resin, centrifugation, and resuspension of the pellet	[190–192]
Bitan	Anti-L1CAM (clone 5G3)	M-270 Epoxy Dynabeads^®^	Centrifugation of plasma or serum samples at 2000×*g*. EV precipitation using ExoQuick, then resuspension in PBS, pH 7.4 + BSA, and incubation with antibody-coated Dynabeads^®^ overnight at 4 °C. Washing of bead–EV complexes and elution or lysis of captured EVs	[47, 134, 169]

NCAM is a neuronal cell adhesion protein involved in cell–matrix and cell–cell interactions, whereas L1CAM is an axonal glycoprotein that plays a critical role in CNS development and its rare mutations cause CRASH syndrome [198]. Both NCAM and L1CAM have been hypothesized to be present on the surface of nEVs and indeed were found at considerable levels on the surface of EVs isolated from cultured rat cortical neurons [51]. Neither NCAM nor L1CAM are exclusively specific to CNS neurons. Thus, researchers using these markers have acknowledged that they enrich nEVs, but do not provide absolute specificity for CNS-originating EVs. Because NCAM is expressed in more non-CNS tissues than L1CAM, after the original papers, most groups have opted to use L1CAM rather than NCAM for the IP of nEVs.

Although L1CAM exists in both soluble and membrane-bound forms and is expressed in several organs and tissues outside the CNS [77, 132], thanks to its abundant expression in CNS neurons, many groups have relied on this marker for the IP of nEVs. A recent review by Gomes and Witwer has summarized the research practices and trends in the separation and enrichment of nEVs using L1CAM as a target marker [65]. To date, dozens of studies have demonstrated the utility of capturing and analyzing nEVs isolated using anti-L1CAM antibodies. Nonetheless, the heterogeneous nature of L1CAM, due to alternative splicing, glycosylation, truncation, and other post-translational modifications [11, 72, 148], prompted Norman et al. to raise concerns regarding the ability of antibodies against this protein to capture bona fide nEVs [132]. To address this concern, they used size-exclusion chromatography (SEC) and density gradient centrifugation fractionation of CSF or plasma using a system designed to separate EVs from soluble proteins and tested if L1CAM co-eluted with common EV markers, such as CD9, CD63, and CD81. They found that the fractions containing EVs were rich in tetraspanins, yet most of the L1CAM co-eluted in non-EV fractions, Therefore, they concluded that L1CAM was not associated with EVs in human plasma or CSF. As a result, they recommended against using L1CAM as a marker for the isolation of nEVs. To test for non-specific binding of α-syn to anti-L1CAM-coated beads, Norman et al. performed immunocapture using recombinant α-syn. In our view, their choice of the recombinant protein was not ideal for this purpose, because competitive blockers present in serum/plasma samples, such as albumin and other highly abundant proteins, were absent.

**Fig. 3:**
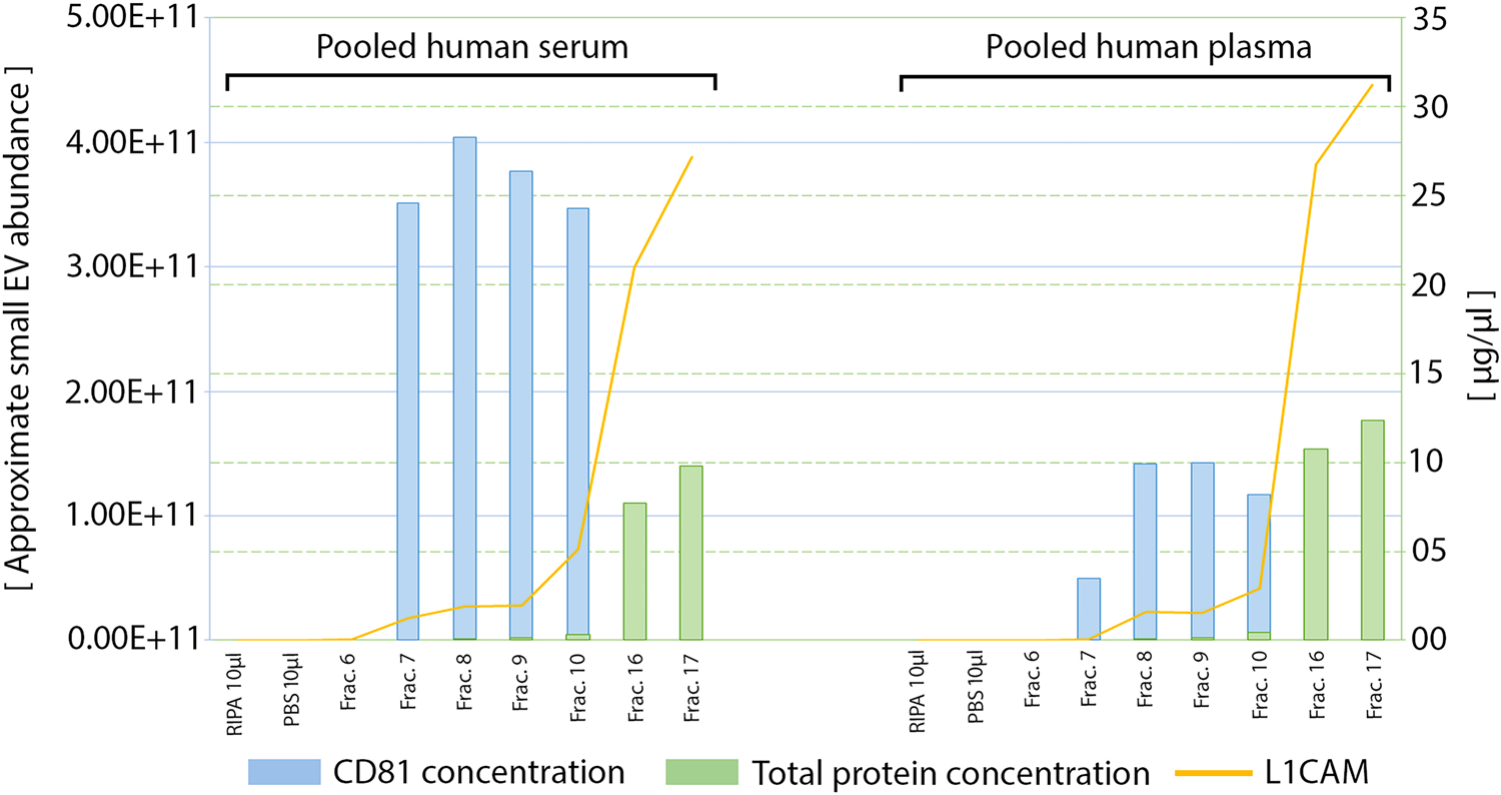
Measurement of L1CAM in SEC fractions containing EVs. L1CAM, total protein, and CD81 were measured in pooled human serum or plasma fractions fractionated using a qEVoriginal size-exclusion chromatography column (Izon Sciences). Fractions were collected, and protein concentrations were measured using a BCA assay and analyzed for the presence of L1CAM and CD81 using ELISA. EVs were eluted in fractions 7–10. Fractions 16 and 17 contained most of the rest of the serum/plasma materials and were diluted 5 × 10^4^ times for L1CAM assay. The graphs represent two independent experiments

Following the report by Norman et al., several groups, including our own, addressed those concerns. We reproduced the fractionation experiments they reported using commercial, pooled human serum or plasma using the same size-exclusion columns designed to separate small EVs from soluble proteins (35 nm, qEVoriginal, Izon sciences). We then assessed the fractions for the presence of L1CAM using a commercial ELISA kit (Human L1CAM ELISA kit, Millipore-Sigma), which is less sensitive than the Simoa assay Norman et al. used. We also measured the concentration of CD81, a proposed canonical exosomal marker, using ExoELISA-ULTRA, CD81 detection (System Biosciences). Our analysis showed that although most of the L1CAM signal was indeed found in the fractions containing free proteins, as reported by Norman et al. [132], all the fractions containing EVs from the serum or plasma were L1CAM positive (Fig. 3). We did not detect any signal in PBS or RIPA buffer, excluding matrix effects [47].

Despite the reassurance by us and other groups that L1CAM could be used for isolation of nEVs, more specific markers have been actively sought after. Recently, Tian et al. identified the synaptic glutamate ionotropic receptor, N-methyl-D-aspartate (NMDA) subunit 2A (NMDAR2A) as a novel marker for isolating CNS-originating EVs from blood [174]. They used a flow cytometry-based method for measuring a combination of markers including L1CAM, NMDAR2A, Aβ40, Aβ42, pS396-tau, and pT231-tau, which helped differentiate among patients with AD, PD, and healthy controls. Moreover, the glutamate receptor subunits 2 and 3 (GluR2/3) were found to be associated with neuronal EVs [51] and targeting these receptor subunits allowed for the immunoaffinity-based enrichment of nEVs from cell culture supernates and human serum [206]. In a different study, Eitan and colleagues developed a multiplex Luminex-based immunoassay to differentiate among EVs originating in erythrocytes, macrophages, and neurons using antibodies targeting the canonical EV marker CD9, the macrophage marker CD68, the microglial marker purinergic receptor P2RY12, and the neuronal marker growth associated protein 43 (GAP43) [182]. The methodologies for the isolation of nEVs used in selected, recent studies and the biomarkers measured in them are summarized in Table 1.

### Astrocyte-originating EVs (aEVs)

Astrocytes are the largest and most prevalent type of glial cells in the CNS. A growing body of evidence suggests that astrocytes are key regulatory CNS cells expressing a wide range of receptors, messenger systems, and channels [1]. Astrocytes play crucial roles in maintaining the BBB, supporting neuronal function by providing structural and metabolic support, and by controlling ion balance. During pathological conditions, astrocytes are triggered by a large variety of stimuli leading to reactive astrogliosis. Emerging evidence suggests that disruption of astrocyte function is associated with dopaminergic neuron loss in PD [24] and the expression of DJ-1 (PARK7), a redox-sensitive chaperone that protects neurons against oxidative stress and cell death, has been shown to be upregulated in reactive astrocytes in patients with PD [14]. Importantly, α-syn released from neurons is transferred to, and accumulates in, astrocytes where it modulates immune functions [106, 162], emphasizing the importance of astrocytes in PD and potentially other synucleinopathies.

Recent studies indicate that astrocytes release a great number of EVs that are involved both in important normal biological processes and in the spread of neuropathology. Protective roles of aEVs in adverse conditions also have been identified [176]. For example, Apolipoprotein D (ApoD)-containing aEVs have been reported to promote the functional integrity and survival of strained neurons, e.g., during increased oxidative stress, by transferring ApoD from healthy astrocytes to neighboring neurons [138]. Other major beneficial roles aEVs include protection from neuroinflammation [82] and neural injury [192]. aEVs also have been reported to stimulate neuronal survival and maturation and increase neuronal excitability [204], which may be advantageous, but also could be detrimental when hyperexcitability is part of the disease process. We are not aware of studies of aEVs in the context of PD or other parkinsonian diseases, but they have been studied in the AD field. Thus, potential adverse effects mediated by aEVs include harboring Aβ42 and ApoE ɛ4 leading to cytotoxicity in neighboring recipient neurons [160]. In addition, aEVs enriched in dysregulated protein cargo, such as β-secretase 1 (BACE-1) and the soluble fragment of amyloid β-protein precursor generated by β-secretase, sAPPβ, have been shown to trigger neuroinflammatory cascades and neurodegeneration [63, 191].

Though aEVs are a promising source of biomarkers and a target for treatment development [44, 157, 204], compared to studies of nEVs, aEVs have been scarcely explored. For immunocapture of aEVs from blood, researchers have used antibodies against GLAST (glutamine aspartate transporter, also called excitatory amino acid transporter 1) (Fig. 1), which has been used to IP and/or validate aEVs is several studies [62, 131, 179, 192], glial fibrillary acidic protein (GFAP) [183, 189], or aquaporin 4 [183]. To our knowledge, no study has compared the specificity or yield of these markers for capturing aEVs. Of these potential markers, GLAST has been used most frequently. Analysis of potential biomarkers in aEVs immunoprecipitated using anti-GLAST antibodies have allowed measurement of disease-associated proteins, including BACE-1, Aβ42, pT181-tau, and pS396-tau (Table 2), which helped differentiate among neurodegenerative conditions [63, 205, 211].

**Table 2:** Selected human protein biomarker studies using CNS-originating EVs from different brain cell types

Disease	Biomarker			
	nEVs	aEVs	mEVs	oEVs
Parkinson’s disease	α-syn, clusterin, Erk, EV number, GSK-3β, JNK, mTOR, NfL, oligomeric α-syn, phospho p38 MAPK, pS129 α-syn, pT181-tau, SNAP-25, STX-1A, tau, total-Akt, t-p38, VAMP-2 [3, 12, 47, 86, 87, 91, 120, 130, 133, 155, 169]	EV number [133]		α-syn, EV number, NfL, pS129 α-syn, pT181-tau [47, 133, 169, 208]
Multiple system atrophy	α-syn, clusterin, EV number, NfL, pS129-α-syn, pT181-tau [47, 86, 87, 133, 169]	EV number [133]		α-syn, EV number, NfL, pS129 α-syn, pT181-tau [47, 133, 169, 208]
Rapid eye movement sleep behavioral disorder (RBD)	α-syn, clusterin [87, 130]			
Dementia with Lewy bodies (DLB)	α-syn, clusterin [87]			
Frontotemporal dementia	α-syn, Aβ42, BACE-1, clusterin, γ-secretase, GAP-43, GDNF, neurogranin, pS396-tau, pT181-tau, sAPPα, sAPPβ, synaptophysin, synaptopodin, synaptotagmin [61, 62, 87]	Aβ42, BACE-1, γ-secretase, GAP-43, GDNF, neurogranin, pS396-tau, pT181-tau, sAPPα, sAPPβ, synaptophysin, synaptopodin, synaptotagmin [61, 62]		
Progressive supranuclear palsy (PSP)	α-syn, clusterin, oligomeric α-syn, tau [86, 87, 120, 133]	EV number [133]		EV number [133]
Corticobasal syndrome	α-syn, clusterin, oligomeric α-syn, tau [86, 87, 120]			
Alzheimer’s disease	Aβ42, BACE-1, γ-secretase, GAP-43, GDNF, neurogranin, NfL, pS396-tau, pT181-tau, sAPPα, sAPPβ, synaptophysin, synaptopodin, synaptotagmin, total-tau [48, 61, 62, 85, 112, 210]	Aβ42, BACE-1, C1q, C3b, C3d, C4b, C5b-C9 TCC, CD59, CD46, CR1, DAF, Factor B, Factor D, Factor I, Fragment Bb, γ-secretase, GAP-43, GDNF, IL-1β, IL-6, MBL, neurogranin, pS396-tau, pT181-tau, sAPPα, sAPPβ, synaptophysin, synaptopodin, synaptotagmin, TNF-α [61–63]	Aβ, pTau [193]	
Mild cognitive impairment	Aβ42, total-tau, pT181-tau, NfL [85, 112, 210]			
Traumatic brain injury (TBI)	Aβ42, neurogranin, NfL, pS396-tau, pT181-tau, total-tau [192]	Aβ42, C3b, C4b, C5b-C9 TCC, CD59, CD46, CR1, Factor D, factor I, fragment Bb, MBL, neurogranin, pS396-tau, pT181-tau [64, 192]		

*Aβ42* amyloid β-protein residues 1–42, *α-syn* α-synuclein, *BACE-1* β-site amyloid precursor protein-cleaving enzyme 1, *Erk* extracellular signal-related kinase, *GAP43* growth-associated protein 43, *GDNF* glial-derived neurotrophic factor, *JNK* c-Jun N-terminal kinase, *mTOR* mammalian target of rapamycin, *NfL* neurofilament light chain, *sAPP* soluble amyloid β-protein precursor, *t-p38* total p38 MAPK, TNF-α tumor necrosis factor α

### Microglia-originating EVs (mEVs)

Microglia are brain-residing immune cells derived from bone marrow elements infiltrating the brain during early neonatal development [76]. They have been characterized extensively during inflammatory and degenerative conditions, whereas their roles in normal brain physiology are less well understood. Genetic and pharmacologic studies suggest their involvement in CNS homeostasis and maintenance, modulating synaptic plasticity, and regulating neurogenesis [4]. Interaction between neurons and glial cells, including microglia, regulates neuronal communication and function and governs selective neuronal vulnerability to disease-specific stresses, ultimately determining neuronal morbidity [56, 168].

Early studies showed that microglial communication with other CNS cells is mediated by both ligand–receptor interactions and soluble factors [10, 20]. More recently, microglia were shown to release large numbers of EVs in both the resting and the activated conditions, which participate in intercellular CNS communication [32]. The cytokine-laden mEVs secreted during stress conditions have been reported to coordinate inflammatory responses across various regions in the CNS [164]. mEVs also have been shown to modulate presynaptic neurotransmission [113]. Nonetheless, despite the importance of microglia in the CNS, the roles mEVs in modulating neuronal and glial functions are largely unknown.

Isolating mEVs from cultured cells, similar to the isolation of EVs from other types of conditioned cell culture media, is relatively straightforward. In contrast, separating mEVs from total blood EVs is challenging due to the lack of specific markers that distinguish them with certainty from those of peripheral immune cells [122]. Myeloid cell-specific CD11b (Fig. 1) [39] and TMEM119 [101] have been used for this purpose yet these markers also may be expressed by other cells. CD11b, the α-chain of integrin receptor CD11b/CD18 (α_M_β_2_), is expressed abundantly on leukocytes and is also found in lung, colon, kidney, bone marrow, lymphoid tissues, monocytes/macrophages, granulocytes, and natural killer cells [50, 146]. TMEM119 has been reported to be more specific to microglia as antibodies against this protein did not stain infiltrating peripheral immune cells [7, 19]. The protein is expressed in microglia two weeks after birth and has been suggested to be a specific microglial marker [7]. However, according to the human protein atlas (proteinatlas.org), TMEM119 is expressed also in the respiratory system, liver, gastrointestinal tract, and lymphoid tissues [171].

Aminopeptidase N (CD13) and monocarboxylate transporter 1 have been suggested as potential markers on mEVs [137, 140], though both may cross-react with other immune cells. Similarly, Iba1, another marker used for isolation and validation of mEVs, is expressed in cell types other than microglia, including Kupffer cells, Hofbauer cells, Langerhans cells, macrophages, and monocytes. The purinergic receptor P2Y12 is another potential microglia-selective marker [214], yet this protein is also expressed at lower levels in the nasopharynx and subsets of cells in the bone marrow and lymphoid tissues. Thus, to our knowledge, a protein expressed exclusively on the mEV surface is yet to be identified and validated. In view of this difficulty, one possibility is to use level-dependent marker expression, e.g., low CD45 and high CD163, to distinguish microglia from monocytes and perivascular macrophages [28], yet this strategy has not been used for specific isolation of blood mEVs and likely will be difficult to use for this purpose.

### Oligodendrocyte-originating EVs (oEVs)

Oligodendrocytes are specialized, large glial cells in the CNS that assemble myelin, a multilayered sheath insulating the electrical signal along axons. Oligodendrocytes wrap themselves around axons and offer trophic support [128]. Oligodendrocyte-mediated myelination of axons requires intense communication between the two cell types [5]. oEVs are important for axon-oligodendrocyte communication, shuttling active biomolecules from the oligodendrocytes to the neurons, promoting fast axonal transport, and maintaining axonal transport in starving neurons [58]. In a co-culture of primary mouse neurons and oligodendrocytes, oEVs have been shown to promote neuronal survival under ischemic conditions, possibly by transfer of superoxide dismutase (SOD) and catalase via oEVs from the oligodendrocytes to the neurons [55].

In the rare synucleinopathy MSA, unlike in PD or DLB, α-syn deposits as glial cytoplasmic inclusions (GCIs) primarily in oligodendrocytes. This phenomenon led us and others to hypothesize that oEVs’ content might provide useful information and potential biomarkers for MSA. Following this logic, Yu et al., tested if the number of oEVs and their α-syn content were altered in patients with MSA compared to those in patients with PD. They used an antibody against the oligodendrocyte marker 2,3-cyclic nucleotide-3-phosphodiesterase (CNPase) for oEV IP [208]. We used a similar strategy for comparing α-syn concentration in nEVs and oEVs as a potential diagnostic biomarker for MSA and PD [47], but utilized a different marker for IP of oEVs, myelin oligodendrocyte glycoprotein (MOG).

### Validation of the cellular origin of CNS-EVs isolated from peripheral biofluids

Though important progress has been made recently in isolating and enriching EVs originating in all four brain cell types from biofluids including serum and plasma, in most cases the cellular origin of these EVs was not tested rigorously. Among the reasons are the dearth of highly specific marker proteins on the EV surface that could be targeted for validation and the availability of the respective antibodies. Other typical issues are the minute amounts of the targeted analytes present in these EVs and the difficulty obtaining sufficient sample volumes in large studies using patient samples. A possible solution is the amplification of nucleic acids, e.g., mRNA or microRNA (miRNA), specific for the cell of origin as validation markers.

To demonstrate that their nEVs originated in CNS neurons, Kluge et al. used large plasma volumes in western blots and showed that the nEV preparations were enriched in synaptophysin and neuron-specific enolase [91]. In a different study, Blommer et al. used fluorescence microscopy to visualize EVs double-immunolabelled for L1CAM and the neuronal marker vesicle-associated membrane protein 2 (VAMP2) [23]. These data support the notion that the two-step nEV-enrichment process, including the initial isolation of total EVs, followed by the removal of the supernate, which contains most of the free L1CAM, allows the capture of L1CAM-positive EVs by IP in the next step.

**Fig. 4:**
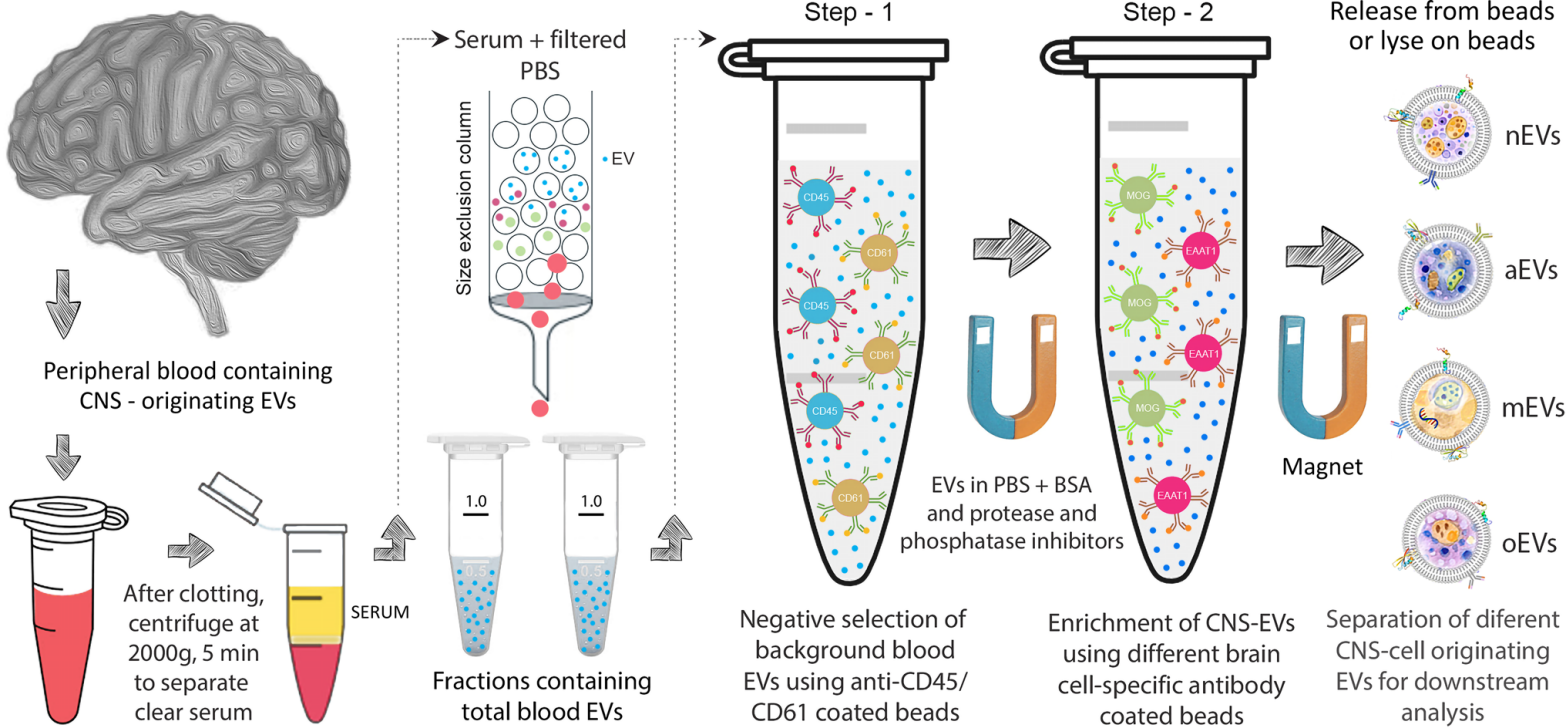
Two-step isolation of CNS cell-specific EVs. EVs originating in the brain are isolated from the serum. In step 1, background blood EVs are separated, followed by the enrichment of different brain cell-originating EVs by immunoprecipitation

Another difficulty is the non-specific binding of EVs to the solid support used. If the non-specific binding is high, it dilutes the signal of the EVs from a specific cell of origin. A recent study by Fu et al. [59] has suggested using the highly hydrated zwitterionic polymer, poly(carboxybetaine methacrylamide) (pCBMA) to alleviate this problem, a strategy that was adopted by Jiang et al., as discussed below [86, 87]. Fu et al. coated magnetic beads with pCBMA via a reversible addition-fragmentation chain transfer (RAFT) process before conjugating the beads to an antibody. They characterized the resulting bead–antibody complex for its antifouling properties by comparing the non-specific adsorption of bovine serum albumin (BSA) to that of Fe_3_O_4_ beads and observed a 90% reduction in non-specific binding. This could be a significant and useful improvement over using magnetic Dynabeads^®^ or other types of beads commonly used for capturing cell-specific EVs. A negative selection step using anti-CD45/CD61-coated beads, as proposed by Ko et al. [94], could offer additional benefits before immunoprecipitating CNS-originating EVs using cell-specific markers to limit background EVs from leukocytes and platelets binding non-specifically to the beads, prior to enrichment (Fig. 4).

## Advances in EV isolation methods

Besides optimization of the magnetic beads, antibodies, and procedures for the isolation of EVs from biofluids, advances in alternative EV isolation and analysis methods, such as microfluidic diagnostic devices, hold promise for future progress in this field. Numerous such devices and technologies have emerged in recent years allowing the detection of biomolecules on micro- or nanoscales [84]. Here we discuss only studies relevant to CNS-originating EVs and refer to the review by Iyer et al. [84] for a more general overview of diagnostic microfluidic devices for EV and biomarker analysis.

Ko et al. have developed a nanofluidics device termed track-etched magnetic nanopore (TENPO) for sorting of CNS-originating EVs [92, 93]. Plasma EVs were labeled using a biotinylated anti-GluR2 antibody and then reacted with anti-biotin magnetic nanoparticles to label nEVs [51, 94]. The TENPO device consists of a polycarbonate membrane coated by a soft magnetic film. The EV-containing fluid passes through the membrane capturing labeled nEVs at the edges of the nanopores, whereas unlabeled EVs flow through. Analysis of captured EVs showed the enrichment of particles with a diameter of ~ 142 nm as assessed by dynamic light scattering (DLS), typical EV size and morphology in scanning electron micrographs, the presence of the exosomal markers TSG101, Alix, and CD9, and the capture marker GluR2 by western blot analysis [93]. nEV isolation using TENPO coupled with off-chip RNA or protein biomarker analysis has been implemented for murine and human plasma and serum for diagnosis of traumatic brain injury (TBI) and can be applied for biomarker analysis in patients with neurodegenerative diseases, such as synucleinopathies. The track etching process used for manufacturing is widely available making TENPO suitable for translation to clinical settings [93]. As with other techniques, cell-specific EV capture depends on the targeted surface markers and the antibodies used for capture.

More recent studies by Ko et al. proposed a novel antibody-based immunosequencing of single EVs, which could allow multiplexed measurements of different proteins in individual EVs [95, 96]. The studies used microfluidics devices for trapping single EVs into a droplet. In the first step, isolated EVs were labeled with a specific antibody-DNA marker, followed by encapsulation into droplets, and finally, in situ PCR was used to amplify DNA barcodes for an imaging-based readout [95, 96]. Combining this multiplexed, single-EV protein-profiling technology with the existing methods for CNS-EV enrichment may allow the identification of the cellular origin of individual EVs. After enrichment of CNS EVs following standard protocols [78], individual EVs could be released from the beads and labeled with marker antibody–DNA conjugates, including isotype controls for baseline correction. These complexes then could be encapsulated into droplets with different barcoded beads, specific for different markers. However, the composition and levels of different proteins vary greatly in individual EVs. Therefore, precise capture of such EVs and highly sensitive methods for content analysis are essential.

Along these lines, Yang et al. reported the development of a novel high-throughput approach to quantify rare EV subpopulations in biological samples [201]. Their droplet-based extracellular vesicle analysis (DEVA) used fluorescent paramagnetic microbeads (*d* = 5.4 µm) functionalized with an anti-human CD81 antibody for EV capture followed by labeling of the EVs with a biotinylated anti-CD81 detection antibody and formation of an enzyme-linked immunocomplex upon addition of a streptavidin–HRP enzyme. Next, the beads were mixed with the enzyme substrate and suspended as aqueous droplets (*d* = 20 µm) in oil resulting in one or zero beads per droplet and one or zero EVs per bead. Each of the generated droplets was inspected for fluorescence in two channels: one for the fluorescence of the bead and one for the fluorescence of the droplet indicating successful capture of a single EV positive for the target protein CD81. The DEVA assay was used to quantify EVs isolated from human iPSC-derived neurons spiked into fetal bovine serum containing 2 × 10^7^ bovine EVs as background. The assay had a limit of detection of 11 EVs/µL and quantified endogenous EVs in human plasma [201], suggesting that it could be useful for detection of rare EV subpoplations, such as CNS-originating EVs in human plasma/serum for biomedical applications. For this purpose, adjustement of the assay would be required to allow detection of CNS cell-specific markers instead of the general EV marker CD81. Multiplexing of the system using different fluorescence-labeled microbeads for each capture antibody could enable simultaneous detection and possibly isolation of multiple CNS cell-orignating EV subpopulations [201].

## Analysis of biomarkers in CNS-originating EVs isolated from blood products for parkinsonian disorders

In the first study analyzing α-syn in CNS-originating EVs, Shi et al. isolated plasma nEVs and reported a significantly higher concentration, ~ twofold, of α-syn in those of 267 patients with PD compared to 215 age- and sex-matched healthy controls (HC), yet a substantial overlap was observed between the groups [153]. The diagnosis of PD was determined clinically and the data were not validated in a separate cohort. This has been a common issue also in follow-up studies, which could be addressed in several ways in the future: (1) by analyzing samples obtained pre-mortem, which later are validated pathologically after the patients pass away if/when such samples become available in sufficient numbers; samples with post-mortem pathological validation are available for AD and other dementias, but are scarce for parkinsonian disorders [47]; (2) by using samples obtained post-mortem alongside with pathological validation, such as those available at the Banner Sun Health Research Institute, Arizona [18]; or (3) validation of the findings in an independent cohort.

Two important technical concerns regarding the study by Shi et al. [153] were that the putative nEVs were immunoprecipitated directly from patients’ plasma and that the anti-L1CAM antibody used for IP was clone UJ127 [38]. The first concern is related to the fact that the concentration of soluble L1CAM in the plasma is orders of magnitude higher than the membrane-associated forms present on the surface of nEVs. When the IP is performed directly from the plasma, the beads likely become saturated with soluble L1CAM, compromising EV capture. For this reason, most subsequent studies have used the two-step process mentioned above, in which all EVs are isolated first from the plasma or serum using polymer-assisted precipitation, and this preparation, from which the majority of the soluble L1CAM has been removed, then is used in the subsequent IP step [54, 78]. The second concern is specific to studies analyzing α-syn in nEVs. Recently, anti-L1CAM antibody UJ127 has been shown to have significant cross-reactivity with α-syn [132], suggesting that the differences observed between the PD and HC groups might have reflected enrichment of EVs that had α-syn attached to their surface. Though this does not detract from the conclusions of the paper, the data supporting these conclusions might have been misinterpreted.

A few subsequent studies also had used anti-L1CAM antibody UJ127 for IP of nEVs from serum or plasma and then measured α-syn in them [130, 155] before concerns have been raised regarding the cross-reactivity of this antibody with α-syn. The studies reported significant differences in nEV α-syn between HC and PD groups [155] and a progressive, cross-sectional increase in nEV α-syn from HC to patients with iRBD to early- and advanced-stage PD [130]. In the study by Niu et al. [130] the nEV α-syn concentrations in patients with PD correlated with motor deficits assessed using the Unified Parkinson’s Disease Rating Scale (UPDRS) III (*r* = 0.29, *p* = 0.04), with the combined UPDRS I + II + III (*r* = 0.36, *p* = 0.01), and with non-motor deficits using the Non-Motor Symptoms Questionnaire (*r* = 0.3, *p* = 0.039) and Sniffin’ Sticks 16-item test (*r* =  − 0.29, *p* = 0.04). Follow-up analysis of 18 early-stage patients with PD after ~ 2 years showed that longitudinal changes, rather than baseline α-syn, were associated with the progression of motor symptoms, though not with non-motor symptom progression [130].

As mentioned above, Yu et al. recently measured α-syn concentrations in nEVs and oEVs isolated from plasma samples of patients with PD and MSA. The anti-L1CAM antibody clone they used for isolation of nEVs also was UJ127 [208]. They reported that α-syn concentrations were slightly higher in patients with PD than in those with MSA, yet the overlap between the groups was high and the separation was low. In all of these studies, the antibody’s cross-reactivity, relatively small numbers of samples analyzed, and lack of validation reduce the significance of the findings.

In other studies, although anti-L1CAM antibody UJ127 was used for IP of nEVs, the subsequent analysis included analytes other than α-syn. Although interpretation may be complicated by the cross-reactivity of the antibody, the findings could be important and guide future studies. Jiang et al. used a combination of α-syn and clusterin concentrations measured in nEVs immunoprecipitated using anti-L1CAM UJ127, yet a unique modification in their technique was the use of in-house-made pCBMA-coated magnetic beads, expected to have reduce non-specific binding of EVs compared to most commercial polymeric supports used for IP [87]. The study included three independent cohorts: (1) the Oxford cohort containing 65 RBD samples, 48 PD, 26 PD with dementia (PDD), 10 DLB (post-mortem cases), 14 MSA, and 31 HC; (2) the Kiel cohort comprising 155 PD samples, 15 PDD, and 113 HC; and (3) the Brescia cohort including 27 PD, 4 PDD, 11 DLB, 65 frontotemporal dementia, 35 PSP, and 45 CBS samples. The combination of α-syn and clusterin separated efficiently patients with PD from those with atypical parkinsonian syndromes [87].

The group followed up on their first study by adding the PROSPECT cohort, containing 36 MSA, 81 PSP, 43 CBS, and 47 HC samples, and expanded the Kiel cohort from 155 to 215 PD samples. They reported that the combination of α-syn and clusterin analyzed in nEVs separated PD from MSA with 91% sensitivity and 64% specificity, and PD from PSP and CBS combined with 100% sensitivity and 95% specificity [86]. These data are highly encouraging and suggest that clusterin should be considered in future biomarker studies of parkinsonian disorders. Nonetheless, in addition to the use of anti-L1CAM antibody clone UJ127, which complicates data analysis, the pCBMA-coated beads Jiang et al. used currently are not commercially available making validation by other groups difficult. Increased plasma nEV α-syn in patients with PD compared to HCs also was reported by Zhao et al. [212], but provided lower separation power (AUC = 0.654, respectively). Overall, the data summarized suggest that nEV α-syn could serve as a biomarker for early diagnosis of PD and other parkinsonian disorders, whereas correlation of the biomarker with motor symptoms was only found by Niu et al. [130] and not in other studies [47, 153].

An improved diagnostic potential for biomarker panels over single-candidate protein markers has been suggested by Agliardi and coworkers, who precipitated total EVs and enriched nEVs by IP using the anti-L1CAM antibody 5G3 from serum samples of 32 patients with PD and 40 HC. The 5G3 clone was raised against human neuroblastoma cell line SK-N-AS and recognizes the extracellular domain of L1CAM though the exact epitope has not been mapped [126]. Quantification of ‘oligomeric’ α-syn using a sandwich ELISA kit (MyBioSource cat n°: MBS730762) yielded a significantly increased signal in patients with PD, whereas the presynaptic soluble N-ethylmaleimide-sensitive-factor attachment receptor (SNARE) complex proteins STX-1A and VAMP2 were reduced in patients with PD compared to HC. Furthermore, negative correlations between ‘oligomeric’ α-syn levels and both STX-1A and VAMP2 SNARE proteins were reported, leading to an increased discrimination power for the combined biomarkers ‘oligomeric’ α-syn/STX-1A (Sensitivity = 85.7%, specificity = 82.5%) and ‘oligomeric’ α-syn/VAMP2 (Sensitivity = 75.0%, specificity = 92.5%) compared to each marker alone [3]. A positive cross-sectional correlation between α-syn concentrations and disease duration was observed in the patients with PD. Though these data are encouraging, general concerns associated with the specificity of antibodies claimed to be specific for oligomers, the relatively low sensitivity of the kit, 0.1 ng/mL, and an absence of detailed information about the kit itself, including standard composition and preparation [79] suggest that the actual identity of the analytes measured should be scrutinized carefully. The relatively small sample numbers and lack of validation in an independent cohort or using post-mortem samples are additional limitations of this study.

In another recent study, Meloni et al. used commercial ELISA kits for measurement of ‘oligomeric *α*-syn’ and ‘aggregated tau’ in nEVs immunoprecipitated using antibody 5G3 from the serum of patients diagnosed clinically with PD (*n* = 70), PSP (*n* = 21), or CBS (*n* = 19) [120]. As might be expected, oligomeric α-syn was higher in PD compared to PSP and CBS, whereas aggregated tau was higher in the nEVs of patients with the two tauopathies. Combination of both biomarkers separated PD from CBS with AUC = 0.902 and PD from PSP with AUC = 0.880. As discussed above, the ‘oligomeric α-syn’ ELISA presumably is based on binding to an antibody selective for oligomeric α-syn, though no details are provided about the identity of the antibody and cross-reactivity with other forms, including monomer and/or fibrillar aggregates might occur. The ‘aggregated tau’ assay is different and more reliable in nature. It uses the anti-human tau antibody 8F10, which binds the C-terminal epitope tau_428-437_, for both capture and detection of the analyte. This configuration ensures that monomers are not detected by the assay, though oligomers as small as a dimer and assemblies as large as fibrillar aggregates, which may contain hundreds of molecules, are detected by this assay. The signal amplitude is higher for larger aggregates because they contain larger numbers of epitope copies, which complicates data interpretation. Nonetheless, this is an important demonstration that measurement of disease-relevant proteoforms can be measured in nEVs.

In our group’s studies [47, 169], we used magnetic Dynabeads^®^ coated with the anti-L1CAM antibody 5G3 or anti-MOG antibody (D-2) for IP of nEVs and oEVs, respectively. To test for potential cross-reactivity with α-syn, we tested the level of α-syn binding to beads conjugated to each of the antibodies, or a control mouse IgG. In all cases, using ECLIA we found similar amounts of non-specifically bound α-syn to the antibody-conjugated beads [47], which were 42–60 times lower than those reported previously for anti-L1CAM antibody clone UJ127 [132]. D-2 is specific for an epitope within the C-terminal extracellular domain of human MOG. We chose MOG because it is CNS myelin-specific and located on the surface of mature oligodendrocytes [89]. Interestingly, our analysis yielded distinct results from those of Yu et al., who also compared α-syn levels in nEVs and oEVs [208]—we found that α-syn concentrations were significantly higher in both nEVs and oEVs from patients with MSA compared to those with PD. These differences were observed in a discovery cohort (50 HC, 51 PD, 30 MSA) and showed a high level of reproducibility in an independent validation cohort (51 HC, 53 PD, 50 MSA). The results allowed constructing a composite biomarker model comprising the α-syn concentration in the nEVs, the ratio between the α-syn concentrations in the oEVs and nEVs, and the total concentration of the EVs in the sample. The model was trained on the discovery cohort and then applied to the validation cohort, in which it separated PD from MSA with AUC = 0.902 [47]. More recently, we found that adding oEV pS129-α-syn, a particularly pathologic form of the protein, to the model improved the separation further to AUC = 0.936 [169].

A potential explanation for the contradictory results in the studies of Dutta et al. [47] and Yu et al. [208] is that although both CNPase and MOG are membrane-bound proteins expressed specifically by oligodendrocytes, CNPase is present on the cytosolic side of non-compact myelin [46, 175] and the intermembrane space of mitochondria [108], which may limit its presentation on the surface of EVs. These data highlight the importance of the marker selection for IP of CNS-originating EVs. Moreover, the difference between the two studies underscores a crucial point discussed in more detail in ‘Omics approaches for identifying novel biomarkers in CNS-originating EVs’: the basis for using a certain marker for IP is the hypothesis that the marker is expressed in sufficient quantities on the surface of EVs originating in the cell of interest and not on the surface of EVs secreted by other cells. Due to the technical difficulty of working with the limited amount of material available in typical EV preparations from patients’ blood samples, to date, few studies have tested this hypothesis.

Recently, Kluge et al. immunoprecipitated nEVs using the anti-L1CAM antibody C-2 (Santa Cruz Biotechnology) from the plasma of 30 patients with PD and 50 HC and measured several biomarkers in these nEVs [91]. Interestingly, in contrast to the studies discussed above [3, 47, 54, 86, 87, 130, 153, 155, 212], they did not find significant differences in α-syn concentrations in the nEVs between the PD and HC groups by using the monoclonal antibody Syn-1, which recognizes an epitope in α-syn_91-99_ [139], possibly because the patients were in relatively early stages of disease (Höhn and Yahr score = 2). However, when they used the rabbit monoclonal antibody MJFR-14-6-4-2 (Abcam, also referred to as MJFR-14 in some publications) in dot blots, they observed significantly higher reactivity in the PD group. This antibody is sold as an “anti-alpha-synuclein aggregate antibody” by the company and has been reported previously to bind selectively to filamentous aggregates of α-syn [149]. Further analysis had shown that it bound α-syn fibrils preferentially, had lower binding to α-syn oligomers, and bound α-syn monomers with even lower affinity [102]. Importantly, Kluge et al. also used a seed-amplification assay in their samples and found signal amplification only in nEVs from patients with PD, providing a highly sensitive means for separating the patients from the HC group [91]. This is the first demonstration of applying a seed-amplification assay in CNS-originating EVs and it suggests that such assays also could be used for the diagnosis of other parkinsonian disorders. The success of Kluge et al. in separating the groups using both the dot-blot and the seed-amplification assays was in a large part thanks to collecting a relatively large volume of blood (15 mL) from each subject. Recapitulating these results in lower sample volumes typically available for specific studies in biorepositories, such as the Parkinson’s Progression Markers Initiative (PPMI), may be difficult, yet the successful demonstration of separation between the groups likely will encourage other researchers to attempt replicating and expanding these findings.

The data presented above strongly indicate that the analysis of multiple biomarkers in CNS-originating EVs, preferably from more than one cell type, can provide highly useful diagnostic and possibly progression biomarkers in blood samples of patients with parkinsonian syndromes. The inclusion of at least two independent cohorts, sufficiently large sample numbers, standardized EV isolation methods, and ideally the pathological validation of patient diagnosis are highly important factors for obtaining significant and reliable findings, ultimately allowing future clinical application of these methods. A summary of human biomarker studies using CNS cell type-specific EVs in parkinsonian disorders is provided in Table 2, which also includes similar studies in AD, mild cognitive impairment, and traumatic brain injury for comparison.

## Omics approaches for identifying novel biomarkers in CNS-originating EVs

The studies discussed above focused on candidate biomarkers for parkinsonian disorders identified mainly based on known pathological changes in these diseases. An exception was clusterin, which was identified by Jiang et al. using a proteomics approach [86]. It is likely that other biomarkers can be discovered in screening studies using similar or other ‘omics’ approaches, such as transcriptomics, metabolomics, and lipidomics. These approaches analyze the composition of EVs in an unbiased manner, potentially leading to the discovery of novel candidate biomarkers [37]. Here, we discuss recent advances in such studies that can be applied to the characterization of EV subtypes and identifying biomarkers for parkinsonian disorders. Detailed reviews of EV isolation methods and sample preparation for such analyses have been published previously [99, 116, 159].

### Markers of EV type

Due to the co-existence of different membranous vesicles in common EV preparations, it may be important to identify markers that distinguish EV subtypes, e.g., exosomes and microvesicles, for the preparation of pure populations before detailed downstream biochemical analysis. In a comparative study of EV isolation methods, Tauro and coworkers identified new biomarkers of exosome biogenesis, trafficking, and release using immunoaffinity capture for proteomic analysis [170]. This application led to the first discovery of the ESCRT-III component VPS32C/CHMP4C and the SNARE protein synaptobrevin 2 in exosomes. Of note, in all the preparations, the isolated EVs were 40–100 nm in diameter and were positive for the exosome markers Alix, Tsg101, and HSP70, suggesting successful enrichment of exosomes [170]. A later extensive study by Kowal et al. [97] showed that proteins previously thought to be exclusive exosome markers—flotillin-1, HSP70, and major histocompatibility complex (MHC) class I and II proteins were present similarly in larger EVs and were not specific to exosomes. Instead, the authors proposed GP96 as a marker for large EVs; actinin-4 and -1, mitofilin, major vault protein, and eukaryotic elongation factor 2 as markers for medium-sized and large EVs; EH-domain-containing 4, a disintegrin and metalloproteinase domain-containing protein 10, and Annexin XI as markers for small EVs of non-endosomal origin; and syntenin-1, tumor susceptibility gene 101, and CD81 as markers of tetraspanin-enriched small EVs, typically considered to be *bona fide* exosomes [97].

Recently, Guan and coworkers presented a method for analyzing simultaneously proteins and metabolites in plasma-derived EVs [67]. Similar to the study by Kowal et al. [97], they confirmed the presence of actinin-4 mainly in the large EV fraction isolated by centrifugation at 20,000 g, whereas the small EV fraction isolated at 100,000 g was strongly enriched in syntenin-1. Proteomic analysis using liquid chromatography–mass spectrometry (LC–MS) highlighted 20 proteins upregulated in the small EV fraction, e.g., complement factor properdin and α1-microglobulin, and 92 proteins upregulated in the large EV fraction, including tetraspanin 32 and magnesium transporter 1. Moreover, 16 metabolites were enriched in small EVs, such as cellobiose and sucrose, and 23 metabolites were enriched in large EVs, e.g., inosinic acid and raffinose. Importantly, the proteins and metabolites identified in the small and large EV fractions were associated with different biological processes and pathways, underscoring the distinct biological functions of these EV subtypes [67]. Altogether, these studies suggest that ‘omics’ analyses are useful for the identification of biological markers that discriminate EVs based on their size and biogenesis pathway. However, strict isolation and validation procedures have to be applied to assure the purity of the analyzed vesicles.

### Markers of CNS cellular origin

In recent years, the identification of biological markers reflecting the cellular origin of EVs, especially those coming from the CNS into the peripheral circulation, has gained increasing interest. Several omics studies focused on the characterization of CNS-originating EV composition aiming to discover novel disease biomarkers or markers of the EVs’ specific cellular origin. To our knowledge, only one such study was performed in the context of parkinsonian disorders. Therefore, we summarize below also studies in related disease and other systems.

Anastasi et al. reported a method combining centrifugation and immunocapture for isolating nEVs from the plasma of four patients with PD and four healthy controls for LC–MS/MS proteomic analysis. After three initial centrifugation steps, plasma sample supernates were incubated in 96-well plates coated with anti-L1CAM antibody UJ127 to enrich nEVs. Subsequent proteomic analysis identified 23 proteins related to PD, including 10 proteins involved in the UPS known to be impaired in PD, and the previously proposed biomarker DJ-1/PARK7 [9, 212]. Other circulating biomarkers of PD, such as gelsolin, serum amyloid P, clusterin, and CXCL12 also were identified in this analysis [9]. Two of these proteins, clusterin, and gelsolin, had been identified in serum EVs of patients with PD in a previous study [87] and gelsolin was reported to be present in Lewy bodies [187]. As discussed above, nEV clusterin recently has been studied together with α-syn and showed promise for differentiating PD from atypical parkinsonian disorders [86, 87].

A proteomics study compared the proteome of CNS-originating EVs from the transgenic SOD1_G93A_ ALS mouse model and non-transgenic control mice. CNS-originating EVs isolated from the extracellular space of whole mouse brains were enriched in MOG and SNAP-25 in wild-type mice, whereas SOD1_G93A_-mouse EVs were enriched in protein disulfide isomerase, an enzyme that has been linked to ALS pathology [13]. Though EVs from specific cellular populations were not isolated, the reduction of the oligodendrocyte marker MOG and the synaptic marker SNAP-25 in EVs from the ALS mice likely reflects the process of neurodegeneration and suggests that these proteins are promising markers for early signs of ALS and possibly other types of neurodegenerative diseases [156].

Lemaire and colleagues isolated EVs from a primary leech microglia culture by ultracentrifugation in combination with either a density gradient or size-exclusion chromatography and identified a signature of six miRNAs, which are potentially characteristic for mEVs using transcriptomics [110]. The isolated mEVs were not tested for surface markers of EV type but TEM micrographs and ultracentrifugation suggested the enrichment of mainly small EVs [110]. Translation of the findings from medicinal leech mEVs to human mEVs has yet to be demonstrated.

The studies discussed above suggest that omics approaches, especially proteomics, are feasible in EVs and may lead to the discovery of new biomarker candidates for neurodegenerative diseases. Nevertheless, omics analyses of EVs have considerable challenges. Typical issues are contamination of EVs by non-specific binding of common biofluid molecules, e.g., immunoglobulins, complement proteins, and lipoproteins, limited sample availability, and the low abundance of disease-relevant proteins [35, 73]. Rigorous validation of EV populations by testing for the presence of established EV markers and the use of consistent and optimized isolation protocols are key to minimizing the contamination of EV fractions [116]. The relatively large sample volumes required for these studies can be overcome by pooling patient samples [88] though this practice may affect negatively the specificity of the findings. The current challenges may be overcome by future development of new methods compatible with smaller volumes [9], and/or by using conditioned media from cultured induced pluripotent stem cell (iPSC)-derived CNS cells instead of patient biofluids.

A persistent challenge is that to date, no comparative studies of different brain cell-type EVs, e.g., obtained from primary cultures of neurons, oligodendrocytes, and astrocytes, have been performed that would allow the definition of unambiguous markers for their respective cell of origin. Alternatively, meta-analyses of existing data from large EV databases such as ExoCarta (http://www.exocarta.org), EVpedia (https://exosome-rna.com/tag/evpedia/), Vesiclepedia (http://microvesicles.org), and EV-TRACK (https://evtrack.org) could help identify markers for distinguishing EVs originating from different CNS cell types.

## EV-associated nucleic acid biomarkers in parkinsonian disorders

Both DNA and RNA are found in EVs and can serve as biomarkers [177, 184]. EV RNA molecules include mRNA, transfer RNA (tRNA), circular RNAs (circRNAs), and ncRNAs. EVs carrying long ncRNA (lncRNA) can be transferred from neurons to the blood and have shown promising results as clinical CNS biomarkers in people suffering from gliomas [33, 152]. Multiple studies identifying non-EV-associated RNA biomarkers of parkinsonian disorders in CSF, serum, plasma, saliva, or urine have been reviewed elsewhere [25, 147]. In contrast, to our knowledge, only one study has examined such biomarkers in CNS-originating EVs to date [213].

Although mRNA and lncRNA are found in EVs, EVs are highly enriched in miRNAs [80] and miRNAs have been the primary RNA biomarkers studied in EVs for parkinsonian disorders, Thus, we focused our analysis on those. We analyzed studies using CSF EVs, serum and plasma EVs that did not attempt to isolate CNS-originating EVs, and the one study that measured a specific lncRNA in nEVs. The methods and results of these studies are summarized in Table 3. Unfortunately, the main conclusion of our analysis is that the quantified miRNAs from CSF [68, 186], serum [15, 29, 74] or plasma [129, 185] poorly overlapped both within and across biofluids, suggesting that rigorous standardization of isolation and analysis methods is necessary before meaningful conclusions can be made.

**Table 3:** Summary of EV-associated miRNAs in Parkinson’s disease

Medium	# Patients	EV isolation methodology (company)	RNA extraction methodology (company)	RNA quantification methodology	Candidate RNAs	RNAs increased in PD	RNAs decreased in PD	ROC analysis	Reference
CSF	PD = 47 HC = 27 AD = 28	SG Differential Ultracentrifugation (NA)	miRNeasy Serum/Plasma kit (Qiagen)	TaqMan miRNA assay qRT-PCR	> 20	miR-409-3p, miR-10a-5p, let-7 g-3p, miR-153	miR-1, miR-19b-3p	^a^AUC = 0.99	[68]
CSF	PD = 40 HC = 40	miRCURY Exosome isolation kit (Exiqon)	miRcury RNA Isolation Kit – Biofluids (Exiqon)	Small RNA sequencing qRT-PCR	Let-7f-5p, miR-4234-5p, miR-27a-3p, miR-151a-3p, miR-125a-5p, miR-30c-5p, miR-511-5p, miR-1911-5p, miR-238-5p, miR-335-5p, Let-7d-5p, miR-101-3p, miR-4418, miR-95-3p, miR-10b-5p	Let-7f-5p, miR-125a-5p,	miR-27a-3p, miR-423-5p, miR-151a-3p	^b^ AUC = 0.96	[150]
Serum	PD = 109 HC = 40	Total Exosome Isolation reagent from body fluids (Invitrogen)	miRNeasy Mini Kit (Qiagen)	qRT-PCR	miR-24, miR-30a-3p, miR-30e-3p, miR-195, miR-223^*^, miR-324-3p, miR-331-5p, miR-338-3p, miR-505, miR-626, miR-15b, miR-16–2-3p, miR-19a, miR-19b, miR-29a, miR-29c, miR-30c, miR-148b, miR-181a, miR-185, miR-221, miR-339-5p, miR-450b-p, miR1294	miR-24 and miR-195	miR-19b	^c^AUC = 0.946	[29]
Serum	PD = 30 HC = 15 AD = 30 VD = 24 VP = 25	ExoQuick (Systems Biosciences)	miRNeasy (Qiagen)	qRT-PCR	Let-7d, miR-1, miR-9, miR-10b*, miR-15b, miR-19b, miR-22*, miR-23a, miR-24, miR-29a, miR-29b, miR-29c, miR-3b, miR-125b, miR-130b, miR-137, miR-142-3p, miR-148b, miR-181c, miR-191, miR-222, miR-324, miR-505	Let-7d, miR-22, miR-23a, miR-24, miR-142-3p, miR-181c, miR-191, miR-222	NA	^e^ AUC = 0.944	[15]
Serum	PD = 72 HC = 31	Differential Ultracentrifugation	TriPure Isolation Reagent (Millipore Sigma)	Small RNA sequencing qRT-PCR	≥ 185	miR-374a-5p, miR-374b-5p, miR-28-5p and miR-22-5p	miR-199a-3p and miR-151a-5p	^f^AUC range (0.700–0.817)	[74]
Plasma	PD = 52 HC = 48	PureExo Exosome Isolation (101Bio)	Exosomal RNA and Protein Extraction kit (101Bio)	qRT-PCR	miR-626, miR-505, miR-181c, miR-331-5p, miR-193-p, miR-196-p, miR-454, miR-125a-p, miR-137	miR-331-5p	miR-505	^d^AUC = 0.898	[202]
Plasma	PD = 7 HC = 34	ExoQuick (Systems Biosciences) exoEasy (Qiagen)	miRNeasy (Qiagen)	Small RNA sequencing	NA	Let-7e-5p, let-7i-5p, miR-652-3p, miR4732-3p, miR-6131, miR-3184-3p, miR-378 g	miR-197-3p, miR-576,-5p, miR-1468-5p, miR-375, miR-211-5p, let-7e-3p, miR-122-3p, miR-94–1, miR-30d-5p, miR-192-5p, miR-93-5p, miR-425-5p, miR-99b-5p	NA	[129]
Plasma	PD = 32 HC = 13	Differential Ultracentrifugation	miRNeasy (Qiagen)	Next-generation sequencing qPCR	NA	lnc_MKRN2-42:1	NA	NA	[185]
Plasma	PD = 30 HC = 30	exoEasy Maxi Kit (Qiagen)	miRNeasy Mini Kit (Qiagen)	qRT-PCR	miR-15b-5p, miR-30c-2-3p, miR-138-5p, miR-338-3p, miR-431-5p, miR-105b-3p, miR-145a-5p, miR-411-5p	miR-30c-2-3p	miR-15b-5p, miR-138-5p,,miR-338-3p, miR-106b-3p, miR-431-5p	^g^AUC = 0.891	[197]
Plasma (L1CAM neuronal EVs)	PD = 93 HC = 85	Direct L1CAM immunoprecipitation	exoRNeasy Midi Kit (Qiagen)	Microarray hybridization qRT-PCR	NA	NA	Linc-POU3F3	^h^AUC = 0.824	[213]

*EV(s)* extracellular vesicle(s), *SG* sucrose gradient, *qRT-PCR* quantitative reverse transcription polymerase chain reaction, *PD* Parkinson’s disease. *AD* Alzheimer’s disease. *VD* vascular dementia. *VP* vascular parkinsonism

^*^Asterisk indicates antisense miRNA

^a^Model based on miR-153 and miR-409-3p

^b^Model based on combinations of miR-10b-5p, miR-151a-3p and miR-22-3p and α-syn

^c^Model based on a combination of the three miRNAs

^d^Model based on miR-505

^e^Model based on let-7d, miR-22*, miR-23a, miR-24, miR-142-3p, and miR-222

^f^Models based on univariate separation of the six miRNAs between stage-specific PD patients and healthy controls

^g^Model based on miR-15b-5p, miR-30c-2-3p, miR-138-5p, and miR-106b-3p

^h^Model based on nEVs α-syn and Linc-POU3F3 and plasma glucocerebrosidase activity

In the one study that isolated nEVs, Zou et al. used the anti-L1CAM antibody UJ127 for IP of the nEVs from the plasma and quantified lncRNAs alongside other biomarkers. The study used two separate RNA amplification techniques (Table 3) to measure the lncRNA, Linc-POU3F3, and Simoa to measure α-syn in 93 PD and 85 HC samples [213]. The authors chose Linc-POU3F3 as it has important functions in the CNS, such as regulation of the Delta1 and Sox1 genes, which are important for neurogenic differentiation of stem cells [30, 31] and have been shown to be stable in serum EVs [104]. Linc-POU3F3 was higher in nEVs in patients with PD whereas nEV-associated α-syn and plasma glucocerebrosidase activity were lower in patients with PD. The lower α-syn levels found contradicted the reports discussed in “Analysis of biomarkers in CNS-originating EVs isolated from blood products for parkinsonian disorders”. The separation between the groups based on the differences in Linc-POU3F3 was low. When all three biomarkers, nEVs Linc-POU3F3 and α-syn, and plasma glucocerebrosidase were combined, the groups separated with AUC = 0.824.

The poor reproducibility in the miRNA studies may be due to the difference in isolation, e.g., polymer-based precipitation vs. ultracentrifugation, and quantification, such as a TaqMan miRNA assay as opposed to small RNA sequencing of miRNAs, in addition to differences in disease stage and age of the participants recruited in different studies. As no study to date has examined miRNAs in CNS-originating EV for differentiating among parkinsonian disorders, one way to strengthen the consensus would be to compare the separation among disease groups between miRNA and protein biomarkers in CNS-originating EVs.

## Conclusions

Identifying and validating sensitive and specific biomarkers for parkinsonian syndromes is urgently needed. Inconsistent readouts of blood-based biomarkers for neurodegenerative disorders, including PD, often reflect the disconnect between the CNS biochemistry and the peripheral blood composition due to the presence of the BBB. CNS-originating EVs cross the BBB and can be isolated from bodily fluids, thus holding tremendous potential as a minimally invasive source of biomarkers. Multiple studies have demonstrated the practicality of using CNS-originating EVs for the identification and measurement of biomarkers for various neurodegenerative diseases, including diagnostic and progression biomarkers for PD and atypical parkinsonian disorders. Nonetheless, practical issues, such as the isolation of EVs originating in specific CNS cell types and validation of the cell type of origin remain to be rigorously addressed. Significant efforts have been made in recent years to address these issues, some of which have been the subject of scientific controversy. Though the interest in finding biological markers that reveal the cellular origin of EVs, particularly those entering the peripheral circulation from the CNS, has grown over the past few years, the scarcity of highly specific marker proteins on the EV surface that could be used for IP, the limited amount of material in most studies, and the availability of appropriate antibodies are among the current challenges.

Notwithstanding these challenges, substantial recent progress has been made in the use of CNS-originating EVs as biomarker sources for neurodegenerative diseases in general, and for parkinsonian disorders in particular, by approaches incorporating antibodies selective for pathological forms of the proteins involved in these diseases, the use of EVs from more than one cell type, and the addition of seed-amplification assays to the workflow. These developments hold promise for the development and validation of sensitive and specific biomarkers that will allow including the correct patient population in clinical trials at early stages when the treatments are likely to be most effective and to monitor the treatment effect objectively without relying on clinical assessment.
